# Dose-related relationship between ergothioneine concentrations and risk of preeclampsia

**DOI:** 10.1042/BSR20231076

**Published:** 2023-08-31

**Authors:** Kwok M. Ho

**Affiliations:** Fiona Stanley Hospital, University of Western Australia and Murdoch University, Perth, Western Australia

**Keywords:** Dose response, Ergothioneine, prevention

## Abstract

The study by Kenny et al. is of considerable importance. They concluded that there was a weak association between the ergothioneine levels and maternal age, and if a threshold was set at the 90th percentile of the reference range in the control population (≥462 ng/ml), only one of these 97 women (1%) developed preeclampsia, versus 96/397 (24.2%) whose ergothioneine level was below this threshold. These results suggest that there might be a dichotomized association between ergothioneine concentrations and preeclampsia; and only a high ergothioneine level over 90th percentile of the control population could be protective against preeclampsia. With the kind supply of the dataset from the authors, further analysis using univariable as well as multivariable analyses were performed while allowing for non-linearity between ergothioneine concentrations and risk of preeclampsia using a 3-knot restricted cubic spline function. The univariable results showed that ergothioneine had a significant non-linear association with preeclampsia and it would start to offer protective effect from 300 ng/ml onward. The results were similar to the multivariable analysis. In addition, the analysis also confirmed that body mass index was significantly associated with an increased risk of preeclampsia.

**Dear Editor,**

The author read the article by Kenny et al. [1] with great interest. They concluded that there was a weak association between the ergothioneine levels and maternal age, and if a threshold was set at the 90th percentile of the reference range in the control population (≥462 ng/ml), only one of these 97 women (1%) developed preeclampsia, versus 96/397 (24.2%) whose ergothioneine level was below this threshold. These results suggest that there might be a dichotomized association between ergothioneine concentrations and preeclampsia, and only a high ergothioneine level over 90th percentile of the control population could be protective against preeclampsia.

With the kind supply of the dataset from the authors, further analysis using univariable as well as multivariable analyses were performed while allowing for non-linearity between ergothioneine concentrations and risk of preeclampsia using a 3-knot restricted cubic spline function (S-Plus® 8.2 for Windows, U.S.A., 2010) [2]. The univariable results showed that ergothioneine had a significant non-linear association with preeclampsia and it would start to offer protective effect from 300 ng/ml onward (Table 1 and Figure 1). The results were similar to the multivariable analysis (Table 2 and Figure 2). In addition, the analysis also confirmed that body mass index (BMI) was significantly associated with an increased risk of preeclampsia [3].

**Figure 1:**
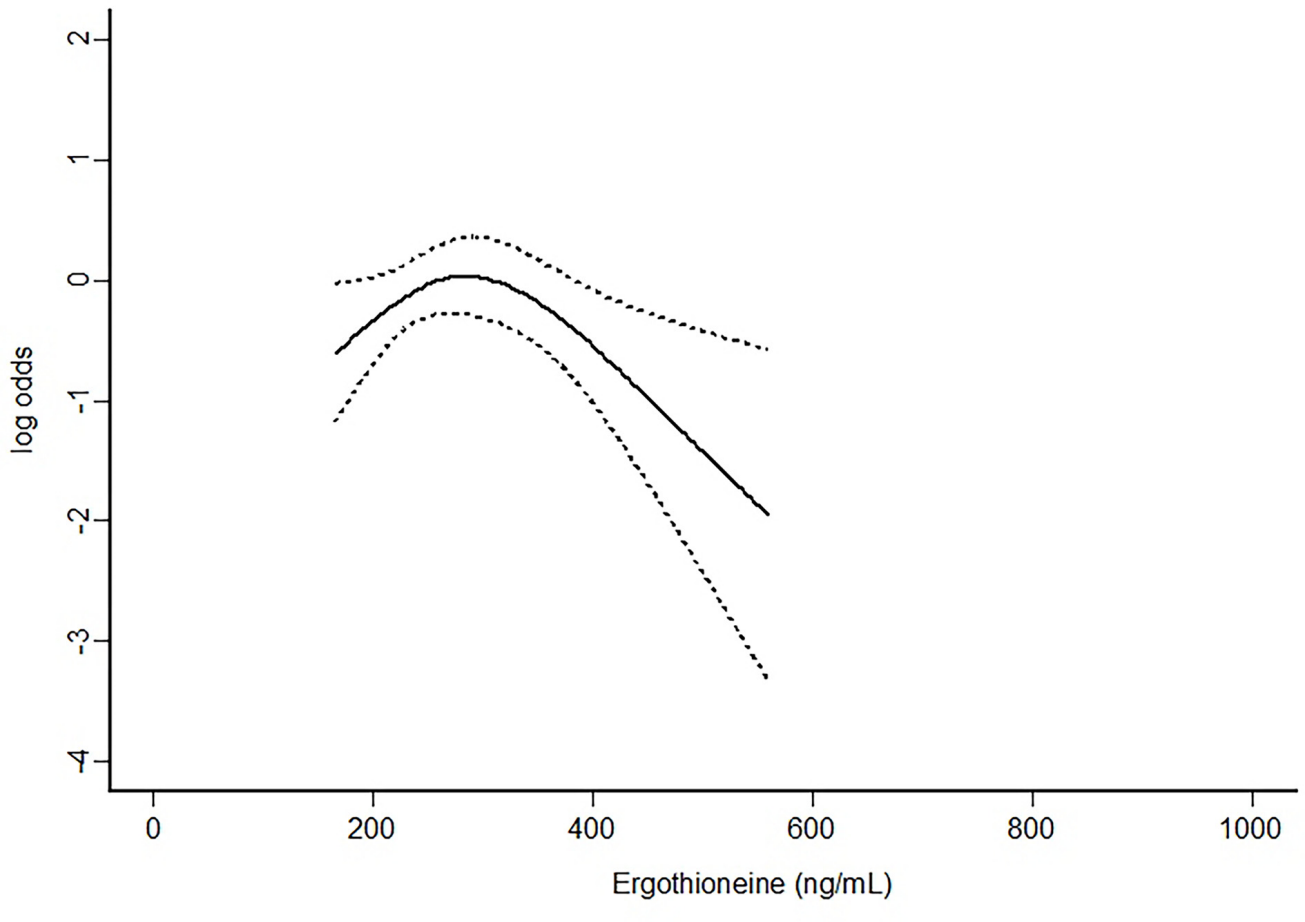
Univariable analysis assessing the association between ergothioneine concentrations and risk of preeclampsia (at preterm or term) allowing for non-linearity using a 3-knot restricted cubic spline function

**Figure 2:**
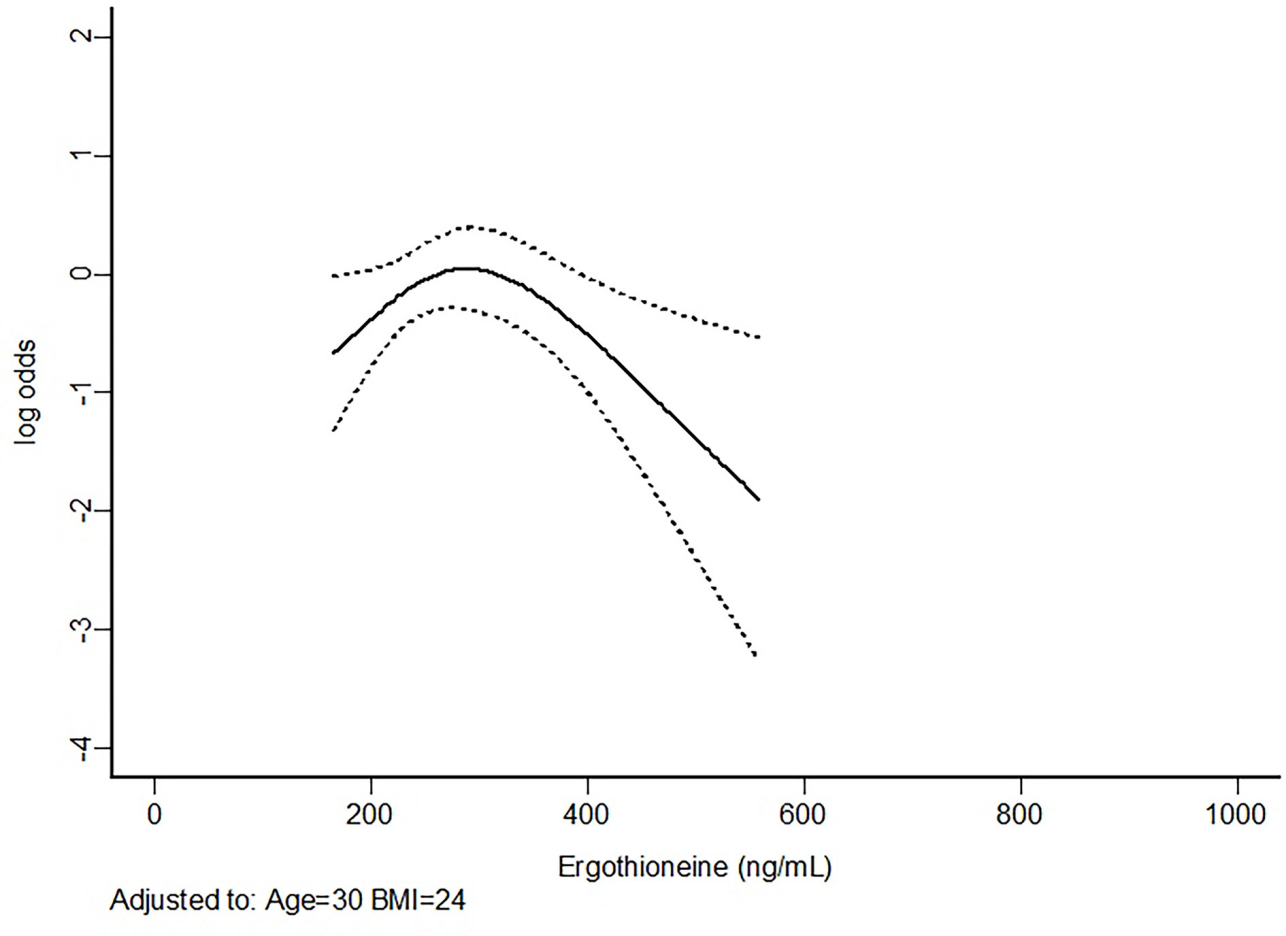
Multivariable analysis assessing the association between ergothioneine concentrations and risk of preeclampsia (at preterm or term) allowing for non-linearity using a 3-knot restricted cubic spline function while adjusting for body mass index (BMI) and age

**Table 1:** Statistical association between ergothioneine concentrations and risk of preeclampsia after allowing non-linearity

Factors in the model	Chi square	Degree of freedom	*P*-value
Ergothioneine	7.14	2	0.0281
Non-linear component of ergothioneine	6.31	1	0.0120
Total	7.14	2	0.0281

**Table 2:** Statistical association between ergothioneine concentrations and risk of preeclampsia after allowing non-linearity and adjusted for age and BMI

Factors in the model	Chi square	Degree of freedom	*P*-value
Ergothioneine	7.14	2	0.0282
Non-linear component of ergothioneine	6.31	1	0.0120
Age	0.02	1	0.8866
BMI	9.62	1	0.0019
Total	16.36	4	0.0026

The results of the study by Kenny et al. have potential important research implications. First, observational studies are important in allowing us to formulate robust testable hypotheses. The study by Kenny et al. provides important foundation to justify conduction of a large observational study to strengthen the causal association between ergothioneine and preeclampsia, preferably involving women from multiple ethnic groups as well as beyond the confine of nulliparous women and singleton pregnancy. Second, if this large observational study can confirm the strength of the effect size of ergothioneine in preeclampsia reported by the current study (absolute risk difference between below and above 90th percentile ergothioneine levels = 23.2%, 95% confidence interval: 17.1–27.7%)[1], a randomized controlled trial (RCT) assessing whether ergothioneine supplementation can reduce risk of preeclampsia will be imminently feasible. Ideally, such RCT should compare placebo with a range of different doses of ergothioneine to identify the best or minimal effective dose, given its good safety records including in pregnant people with a no-observed-adverse-effect level (NOAEL) of 800 mg/kg body weight per day [4]. As BMI and previous preeclampsia will affect a person's underlying risk to develop preeclampsia during pregnancy [3], there is a potential interaction between these risk factors and the efficacy of ergothioneine in preventing preeclampsia. Therefore, stratifying participants by these risk factors in a RCT will be useful in confirming any potential differential protective effects of ergothioneine for various subgroups of pregnant women. Until then, it would be premature to monitor pregnant women’s blood ergothioneine levels and to advise them to modify their diets or take ergothioneine supplementation to prevent preeclampsia.

## Open Access

Open access for this article was enabled by the participation of The University of Western Australia in an all-inclusive *Read & Publish* agreement with Portland Press and the Biochemical Society under a transformative agreement with CAUL.

## Abbreviations

BMI: body mass index
NOAEL: no-observed-adverse-effect level
RCT: randomized controlled trial
